# How to Obtain Laundry Hands

**Published:** 1916-02-12

**Authors:** 


### How to Obtain Laundry Hands.

*To the Editor of* The Hospital.

Sir,—I was talking to the matron of a hospital which shall be nameless, and she told me that she had had great difficulty in obtaining laundry hands prior to the new rules for the restriction of the liquor traffic coming into force, from which time some of the girls employed in the local bars volunteered help in the laundry in the restricted hours, and she accepted, to the mutual benefit of the institution and the girls. This seems to be a very good idea for hospitals throughout the kingdom, and I send it along for what it is worth.—Yours faithfully,

February 2, 1916. Laundry.

